# Advances in Biomechanics-Based Motion Analysis

**DOI:** 10.3390/bioengineering10060677

**Published:** 2023-06-02

**Authors:** Christina Zong-Hao Ma, Zhengrong Li, Chen He

**Affiliations:** 1 Department of Biomedical Engineering, The Hong Kong Polytechnic University, Hong Kong SAR 999077, China; 2 Research Institute for Smart Ageing, The Hong Kong Polytechnic University, Hong Kong SAR 999077, China; 3 School of Mechanical Engineering, Tongji University, Shanghai 200082, China; lizhengrong@tongji.edu.cn; 4 Institute of Rehabilitation Engineering and Technology, University of Shanghai for Science and Technology, Shanghai 200093, China; hechen@usst.edu.cn

Motion patterns in humans have been closely associated with neurological/musculoskeletal/behavioral/psychological health issues and competitive sports performance. Recent decades have witnessed the development of a number of motion capture and analysis techniques to assist professionals in quantitatively evaluating motion patterns. However, current assessments still mainly rely on the professionals’ experience, questionnaires or scales, and functional tests. As a result, some pathological or elite athletes’ motion patterns remained unclear. Moreover, the in-depth biomechanical/neuromuscular mechanisms of motion patterns are poorly understood. Therefore, in this Special Issue, we have assembled 23 research articles and review papers on the state-of-the-art advances in motion analysis from fundamental in vitro cell [1] and cadaveric studies [2] to in vivo experiments on human subjects. These studies have either applied validated biomechanical models and neuromuscular analyses to answer unresolved clinical/sports-related questions or focused on the development of novel motion analysis methods. We expect this Special Issue to shed light on future research and developments in biomechanics and motion analysis.

## 1. Evaluation of Motion Patterns Using Validated Biomechanical Analysis

Biomechanical motion analysis is generally based on two types of models: multibody models and finite element models (FEMs) [3]. A multibody model refers to a set of rigid bodies connected by joints; inverse dynamics are normally incorporated to calculate joint kinetics from the measurable kinematics of body segments [4]. In contrast, FEMs reconstruct internal strain, stress, or deformation in flexible bodies based on continuum mechanics theories [3,5]. These validated models have been instrumental in exploring the motion patterns in specific patients/athletes and examining the effects of specific interventions/treatments on motion patterns. The analyzed body parts range from global posture, balance, gait, or sports performance to localized trunk, upper-limb, or lower-limb joint motions.

Regarding global motion analyses, validated multibody models have been used to quantify postures in healthy adults, gait initiation in patients with Parkinson’s disease, walking patterns in pregnant women, running performance, and swimming performance. Huthwelker et al. [6] quantitatively measured the spine postures in healthy adults of different age and gender groups, serving as reference data for studies of abnormal spine postures. The freezing of gait is common in patients with Parkinson’s disease and may lead to falls; thus, Palmisano et al. [7] investigated underlying balance control in gait initiation and identified that the center of pressure parameters, rather than the center of mass parameters, could be related to the freezing of gait. Li et al. [8] investigated the effects of different shoe-heel heights on pregnant women’s walking balance, providing new insights on reducing fall risks in this population. Fadillioglu et al. [9] compared running patterns in novice runners vs. expert runners, and identified the key spatiotemporal and kinematic parameters indicating better running performance. In addition, Fernandes et al. [10] conducted a comprehensive review on whether swimming performance is related to kinematic parameters, i.e., intracycle velocity variations.

Regarding the motion analyses of localized body components, both validated multibody models and FEMs have been used. Using multibody models, Herteleer et al. [11] continuously monitored shoulder joint angles in patients after surgeries of humerus fractures, and examined the effects of different rehabilitation protocols, i.e., early postoperative mobilization vs. immobilization, on the shoulder joint motions. Similarly, Kwak et al. [2] compared knee joint kinematics following two different protocols of total knee arthroplasty to evaluate the effectiveness of the treatments. However, when some newly proposed interventions cannot be conducted directly on human subjects due to ethical reasons, FEMs can help simulate how interventions may cause changes in specific biomechanical indicators in vitro and simulate the possible clinical outcomes. Giordano et al. [12] used FEMs to examine mechanical properties within the femur (such as stress distribution) by simulating different constructions of implants for treating femur head fractures, and evaluated the treatment effects of different implant construction methods. Similarly, Wong et al. [13] used FEMs to evaluate the stress of different thoracolumbar reconstruction constructs on proximal junctional levels, providing insights on the optimal selection of reconstruction constructs to treat thoracolumbar burst fractures and minimize postoperative complications. In addition, Nispel et al. [14] reviewed the contemporary use of coupled multibody models and FEM simulations to analyze both the holistic biomechanics of the spine and the stress distribution within flexible components (e.g., intervertebral discs), providing a more comprehensive view of facilitating the evaluations and diagnoses of spine-related health issues.

## 2. Evaluation of Motion Patterns Using Validated Neuromuscular Analysis

The in-depth analysis of surface electromyography (sEMG) signals can also be used to explain abnormal motion patterns. He et al. [15] investigated how Schroth exercises, one of the commonly used training methods for patients with adolescent idiopathic scoliosis in clinical settings, activate the paraspinal muscles in concave and convex sides; the findings provide evidence for the effectiveness of this treatment. Son et al. [16] analyzed the sEMG signals of neck, shoulder, and arm muscles during dentists’ daily occupational tasks, and found that the repetition of one task causes muscle fatigue, a finding which supports the importance of rest for reducing occupation-related musculoskeletal disorders. By examining elbow flexor sEMG signals in patients after spinal cord injuries (SCIs) vs. healthy controls, Li et al. [17] found that both the muscle fiber conduction velocity (indicating muscle properties) and the sEMG–force relationship (indicating central neural drive) had been altered after SCI. These applications of validated neuromuscular analyses have complemented biomechanical analyses in advancing the assessment and management of motor function impairments.

## 3. Methodological Optimization and Development in Motion Analysis

To meet the huge demands for wearable motion capture and remote motion analysis in healthcare sectors [18,19,20,21], new trends are emerging to optimize existing motion analysis models or combine them with the novel statistical, machine learning, or deep learning algorithms. Li et al. [22] proposed the use of multivariable linear regression models and a composite index, which was derived from the most significant differences in patients with anterior cruciate ligament deficiency (ACLD) vs. healthy controls, to facilitate the clinical diagnosis of ACLD. Zhao et al. [23] proposed a new model of using only the easily available anthropometric data (i.e., leg length, body weight, and walking cadence) to estimate vertical stiffness in hip and knee joints, providing alternative insights for gait analysis. Human ankle subtalar and talocrural joint motions are difficult to quantitatively measure in outdoor environments; therefore, Agudelo-Varela et al. [24] proposed a wearable device using a new statistical method of angle calculation. Machine/deep learning algorithms have further facilitated marker-free motion capture and analysis. Using machine learning algorithms, Haufe et al. [25] found that the gait events could accurately be determined by as few as two lower-limb muscles’ sEMG signals in patients with Parkinson’s disease. Sikandar et al. [26] used deep learning algorithms to classify walking speeds based on two-dimensional marker-free video images. Similarly, Tang et al. [27] attempted to estimate joint moments and power using video data and deep learning algorithms; however, differences existed when comparing marker-free and marker-based estimates, which indicated that their marker-free approach could be further improved to identify the joint centers/center of segment mass more accurately. In addition to video images, Wang et al. [28] utilized motion data collected by two inertial measuring units (IMUs) to identify students’ classroom behaviors using deep learning algorithms. Similarly, Xia et al. [29] used IMUs and thin-film force sensors in hand exoskeletons designed for stroke survivors, enabling intention recognition based on the biomechanical data collected using the deep learning algorithms.

## 4. Conclusions

Collectively, the studies presented in this Special Issue have used various validated biomechanical models or proposed novel methods of motion analysis to gain new insights into health-related problems and sports performance. As the editors of this Special Issue, we look forward to the continuous efforts of applying novel biomechanics-based motion analysis to support clinical practice and overcome any unsolved challenges. We expect that further steps are needed to translate the methodological developments of motion analysis methods into broader applications.
